# Modeling Extracellular Matrix Reorganization in 3D Environments

**DOI:** 10.1371/journal.pone.0052509

**Published:** 2013-01-14

**Authors:** Dewi Harjanto, Muhammad H. Zaman

**Affiliations:** Department of Biomedical Engineering, Boston University, Boston, Massachusetts, United States of America; University of Edinburgh, United Kingdom

## Abstract

Extracellular matrix (ECM) remodeling is a key physiological process that occurs in a number of contexts, including cell migration, and is especially important for cellular form and function in three-dimensional (3D) matrices. However, there have been few attempts to computationally model how cells modify their environment in a manner that accounts for both cellular properties and the architecture of the surrounding ECM. To this end, we have developed and validated a novel model to simulate matrix remodeling that explicitly defines cells in a 3D collagenous matrix. In our simulation, cells can degrade, deposit, or pull on local fibers, depending on the fiber density around each cell. The cells can also move within the 3D matrix. Different cell phenotypes can be modeled by varying key cellular parameters. Using the model we have studied how two model cancer cell lines, of differing invasiveness, modify matrices with varying fiber density in their vicinity by tracking the metric of fraction of matrix occupied by fibers. Our results quantitatively demonstrate that in low density environments, cells deposit more collagen to uniformly increase fibril fraction. On the other hand, in higher density environments, the less invasive model cell line reduced the fibril fraction as compared to the highly invasive phenotype. These results show good qualitative and quantitative agreement with existing experimental literature. Our simulation is therefore able to function as a novel platform to provide new insights into the clinically relevant and physiologically critical process of matrix remodeling by helping identify critical parameters that dictate cellular behavior in complex native-like environments.

## Introduction

Cells *in vivo* are often surrounded by extracellular matrix (ECM), a complex network of glycosaminoglycans, adhesion proteins, and structural fibers showing tissue-specific mechanical and structural properties. Even epithelial cells, which are typically found with only their basal layer in contact with the basement membrane, will encounter a three-dimensional (3D) matrix environment when migrating during processes such as wound healing or metastatic cancer. Varying various aspects of ECM, including network structure [1] and mechanical properties [2], have been demonstrated to impact cell behavior. Matrix dimensionality has also been shown to regulate cell fate [3]. Increasing evidence from literature suggests that two-dimensional (2D) ECM models are inherently limited in their scope to capture the ability of cells to form adhesions in three dimensions, which can significantly affect signalling and mechanotransduction responses [3]–[5]. Therefore, it is critical that we use 3D systems to study cell-matrix interactions to gain more physiologically-relevant insights.

Among cellular processes inherent to native 3D environments is the fundamental process of ECM remodeling. Matrix remodeling is a dynamic, ongoing process in which cells may deposit new matrix components, break down existing matrix proteolytically with matrix metalloproteases (MMPs), or pull on the matrix with their actomyosin machinery [6]. ECM remodeling is particularly important in 3D environments since cells are more likely to encounter steric obstacles to movement in 3D than when seeded on flat substrates. This remodeling activity has far-reaching implications on cell migration [7], development [8] and various pathological conditions including cancer [8] and various heart diseases [9].

Collagen, the most important structural component of ECM and a readily available biomaterial, is frequently used in 3D matrix models, in part because of the ease with which the physical and chemical properties of the material may be modulated. For instance, depending on the concentration and pH used to gel collagen, the matrix pore size and fiber size can be altered with a corresponding adjustment in tensile properties [10]. In order to quantify matrix architecture, confocal reflectance microscopy (CRM) has been used to investigate collagen structure [10]–[13], fibrillogenesis [14], and how cells interact with the ECM [15], [16]. Previous studies from our group [17] using CRM have explored how two different prostate cancer cell lines, LNCaP cells and DU-145 cells, over time altered the structure of 3D matrices of varying collagen density to evaluate the effect of the initial ECM structure and mechanical properties. Compared to the less invasive LNCaP cells [18]–[21], DU-145 cells modified matrices to yield denser microenvironments, depositing more collagen in the gels initially containing low amounts of ECM, and degrading less of the matrix in gels with high collagen content. In this current work, we extend our knowledge of matrix remodeling in 3D through simulation, using a 3D Monte Carlo lattice model.Our goal is to develop a novel platform for understanding cell-matrix interactions and to provide mechanistic and quantitative insight into how the interplay between matrix properties and cell phenotype affects matrix remodeling.

It is important to note that despite the importance of matrix remodeling *in vivo*, computational and mathematical studies of ECM remodeling have been relatively limited. The few models that do exist have focused largely either on the molecular level or at the tissue level. Israelowitz et al. simulated the structure of type I collagen from the amino acid sequence to determine how the protein interacts with other collagen molecules to form higher order structures – fibrils, fibers, and eventually 3D matrices – by looking at intermolecular interactions and minimizing the energy state [22]. Another approach that has been taken is the use of Brownian dynamics. To simulate the assembly of 3D actin networks, Kim et al. used the Langevin equation to model how actin monomers, filaments, and actin cross-linking proteins interact, implementing stiffnesses in the components in varying orientations; they then evaluated the pore size, fiber diameter, and isotropy of the resulting structure, finding bundled fibers and perpendicular cross-links [23]. While the system models actin, a cytoskeletal protein, rather than ECM components, the principles driving network assembly remain the same. This model was later expanded on to examine the viscoelastic properties of such self-assembled actin networks, performing segment-tracking rheology and bulk rheology *in silico*, and then comparing the results to what was observed experimentally [24]. Another molecular-level model has focused on the problem of matrix remodeling rather than assembly, simulating ECM degradation and synthesis using the kinetics of a proteinase/transglutaminase cycle [25]. In this model, based on a system of ordinary differential equations, the ECM converts between insoluble matrix, which is produced by cells in the model at a constant rate, and soluble proteolysis fragment states, with the levels of the soluble fragments regulating proteinase expression.

Simulations of ECM remodeling in physiological contexts using continuum approaches at the tissue-level have also been developed. For instance, Driessen et al. modeled collagen fiber remodeling in the arterial wall, examining how applying mechanical load to the arterial wall, simulated as a tube consisting of an incompressible, fiber-reinforced composite, affected the orientation of the embedded collagen fibers and pressure distribution within the wall [26]. Ramtani et al. examined contraction of the matrix by fibroblasts, simulating the ECM as an adaptive-elastic material, and studying how matrix rigidity affects the extent of cell contraction [27]. Cell-matrix interactions have also been modeled in the context of wound healing using a mechanochemical framework, with cells, ECM density, and growth factors obeying a conservation law, and with the ECM modeled as a linear, isotropic, viscoelastic material [28]. In the context of tumor growth, matrix remodeling has been simulated with a multiphase model that defines volume ratios of tumor cells, healthy cells, and ECM [29]. Cells proliferated and died at rates dependent on the volume ratio of the matrix, with tumor cells being less sensitive to matrix availability, and secreting more matrix or fewer MMPs [29].

In general, the matrix remodeling simulations and models have focused largely at the continuum level, or at the two extremes of molecular or whole tissue length scales, with little or no attention to the intermediate length scale. In this paper, we report a novel model of intermediate scale that explicitly defines cells and the matrix, and accounts for properties of both. In addition, while these existing modeling approaches are highly informative, few, if any, have explicitly examined matrix remodeling in the presence of cells in 3D.

The model developed and presented in this study aims to contribute to our understanding of this important biological phenomenon. Our model provides detailed insights into both the structure of the matrix and the distribution and activity of cells within the matrix, while also providing data that is directly comparable to published results from cellular level experiments. Furthermore, we have created a model that is defined in three dimensions, and is therefore able to provide more relevant physiological insights than 2D models. We are also able to predict different remodeling behaviors by incorporating cell phenotype information and cell density. Our model is therefore a novel platform for a variety of cellular systems and also enables us to identify the specific aspects of cellular properties that contribute to the remodeling observed in normal healthy tissues and in pathology.

## Results

### Model Overview

**Figure 1:**
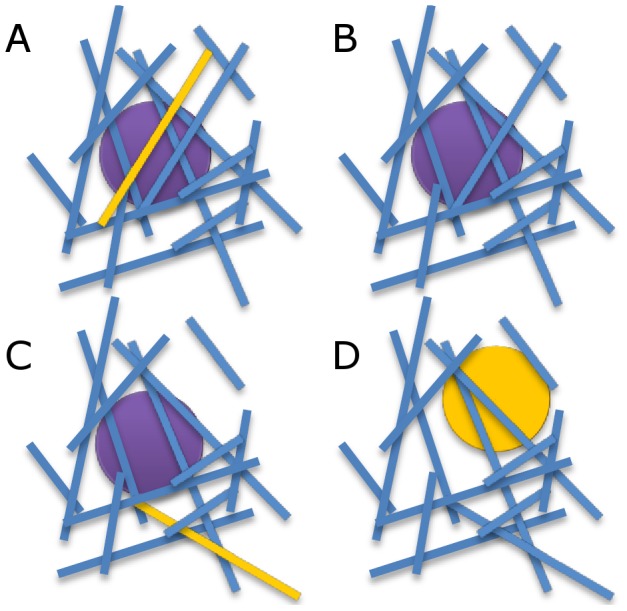
Cells are allowed to remodel and move through the environment. Three types of remodeling behavior are possible in the model: (*A*) Deposition of new ECM fibers; (*B*) degradation of fibers; and (*C*) fiber realignment. (*D*) Cells are also allowed to move through the matrix.

The goal of our model is to create a novel platform for understanding cell-matrix interactions and to provide a detailed mechanistic and quantitative basis for existing experimental results showing how collagen content and cell phenotype influences matrix remodeling. To do so, we used a Monte Carlo lattice model to simulate 3D environments, drawing heavily on our experimental work in this area [17]. In this model, cells are allowed to migrate and to remodel a collagenous matrix, represented by a random assortment of line segments in three dimensions, in one of three ways: fiber degradation, realignment, or deposition (**Figure 1**). The likelihood of each of these possible actions by a cell is defined by a probability function dependent on the number of fibers within a specific radius of each cell, or the local fiber density. The probability of movement is implemented with a Gaussian curve to model the balance between traction and adhesion forces observed experimentally [30]–[32]. Migration in our simulation is accompanied by matrix remodeling in order to accommodate the cell in its new position.

**Figure 2:**
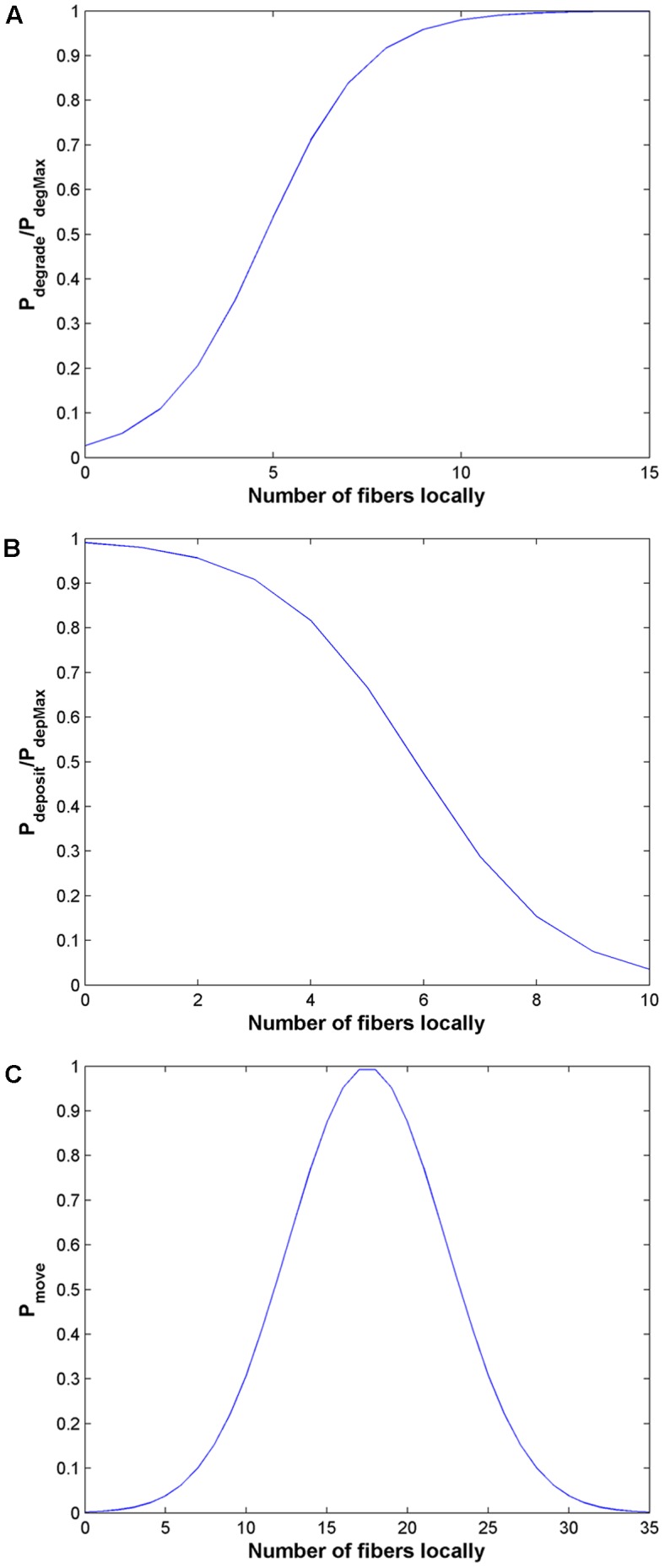
Example probability distributions as a function of local fiber density. Probabilities for degradation and deposition are normalized by maximum probabilities (P_degMax_ and P_depMax_, respectively, which are constants that are determined by cell type) for each form of remodeling. (*A*) Degradation – fibers are more likely to be degraded if local to a cell there are many fibers; (*B*) deposition – new fibers are more likely to be deposited if local to a cell there are relatively few fibers; and (*C*) motility – it is well-established in the literature that cells have a biphasic relationship with ECM ligand availability, and we have implemented the probability of movement accordingly with a Gaussian function.

Cells that do not move in a given timestep are also allowed to remodel the matrix. The probability P_degrade_ is used to determine if a close fiber will be degraded by a cell. The probability function selected (see Methods) was chosen such that there would be a high probability of fiber degradation in the presence of a high local fiber density and a low probability of fiber degradation in the presence of a very low fiber density. There is also a probability that nothing will happen to each close fiber, P_nothing_, and a probability of fiber realignment, P_align_. If the number of fibers close to a cell falls below a certain threshold, there is a probability P_deposit_ of new fiber deposition, again a logistic function dependent on local fiber density. Example probability distributions are shown in **Figure 2**.

Different cell phenotypes are modeled by varying the various probabilities and thresholds governing motility and remodeling. The model outputs the fraction of matrix occupied by collagen fibers at specified time points. The simulations were run to simulate one week of biological time. The algorithm is discussed in further detail in the Methods section.

### Acellular matrices of different fiber number show varying structural properties

**Figure 3:**
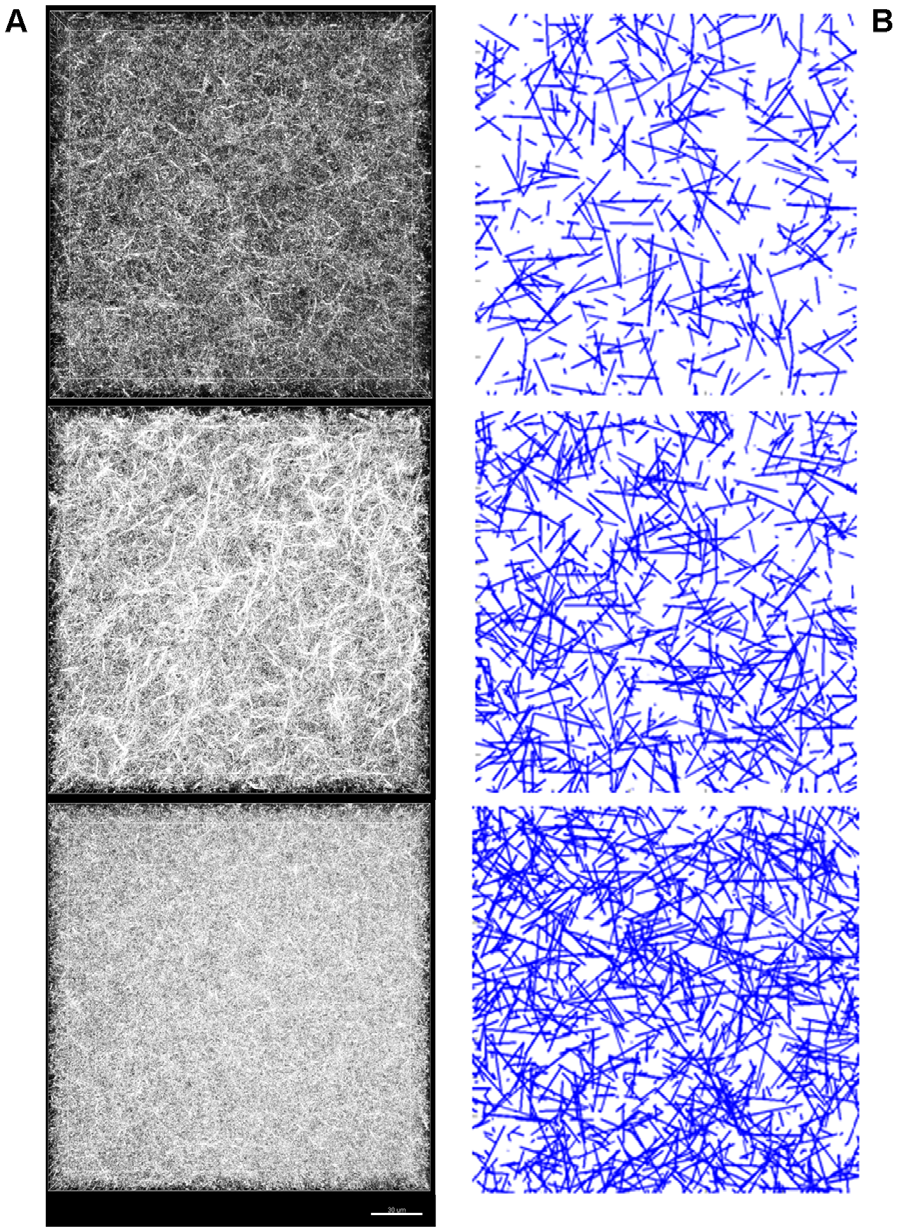
Comparison of experimental and simulated matrices. (*A*) CRM images of 2, 3, and 4 mg/mL collagen gels, from top to bottom (originally shown in [17]); and (*B*) cross-sections of simulated matrices containing 1200, 2000, and 3000 fibers per lattice, from top to bottom. The CRM images show a view of a whole stack of acquired images from above, while the images from the model depict cross-sections of the matrices, which may give the appearance of there being a significant difference in fiber density between the two.

**Figure 4:**
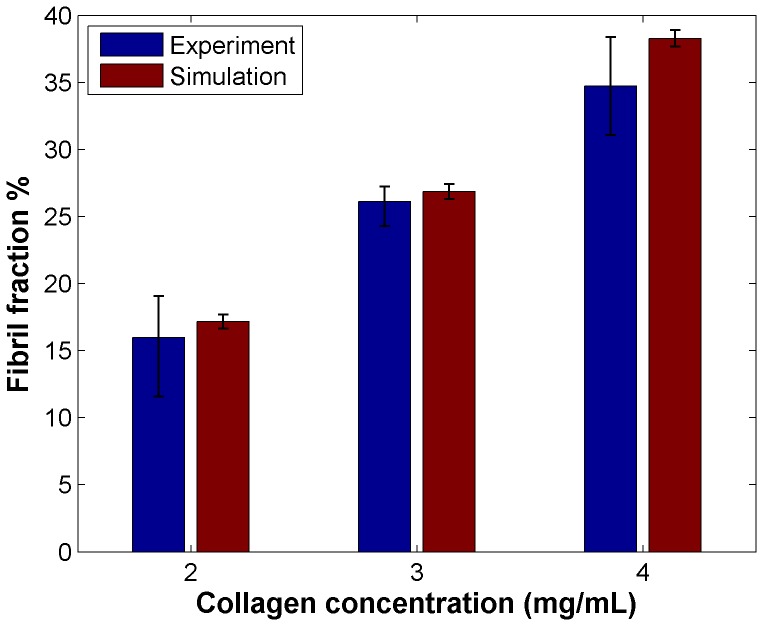
Comparison of fibril fraction for matrices of varying collagen concentration after one week without cells from experiments and simulations. Experimental data was originally reported in [17].

First, gels were simulated without cells to compare the results obtained for the same condition experimentally and to validate the chosen procedure of generating random matrices. Representative images of the simulated matrices with varying fiber number (1200, 2000, and 3000 in a 75×75×75 lattice) are shown alongside experimental CRM images of corresponding collagen gels (2, 3, 4 mg/mL) for comparison (**Figure 3**). The results of the simulated matrices match well with the CRM images, with denser matrices showing smaller pores and a higher fraction of fibers, as would be expected when a given volume contains a higher number of fibers. To compare matrices quantitatively, we used the structural metric of fibril fraction, or the fraction of area occupied by collagen fibers. Fibril fraction was chosen since as it is frequently reported in the literature [17], [33], and is a trackable, physiologically-relevant metric of matrix remodeling. The simulated matrices also provide good agreement with the experimental gels quantitatively, as we find higher fibril fractions (**Figure 4**) with higher collagen content. Quantitatively, simulations and experiments for a given collagen concentration produced data that were not significantly different (p>0.05), while comparisons between distributions of fibril fractions of varying collagen concentration were significantly different in both experiments (p<0.05 for all comparisons, e.g. 2 mg/mL versus 4 mg/mL) and simulations (p<0.01 for all comparisons). The simulated matrices did not evolve over time (data not shown), as was seen experimentally [17], since all matrix remodeling is due to cells. A critical assumption of the model is therefore that the collagen matrix does not inherently change in the absence of cells, an assumption that is corroborated by experimental observations [17].

### Model successfully recapitulates the remodeling behavior observed by two different cell lines

**Figure 5:**
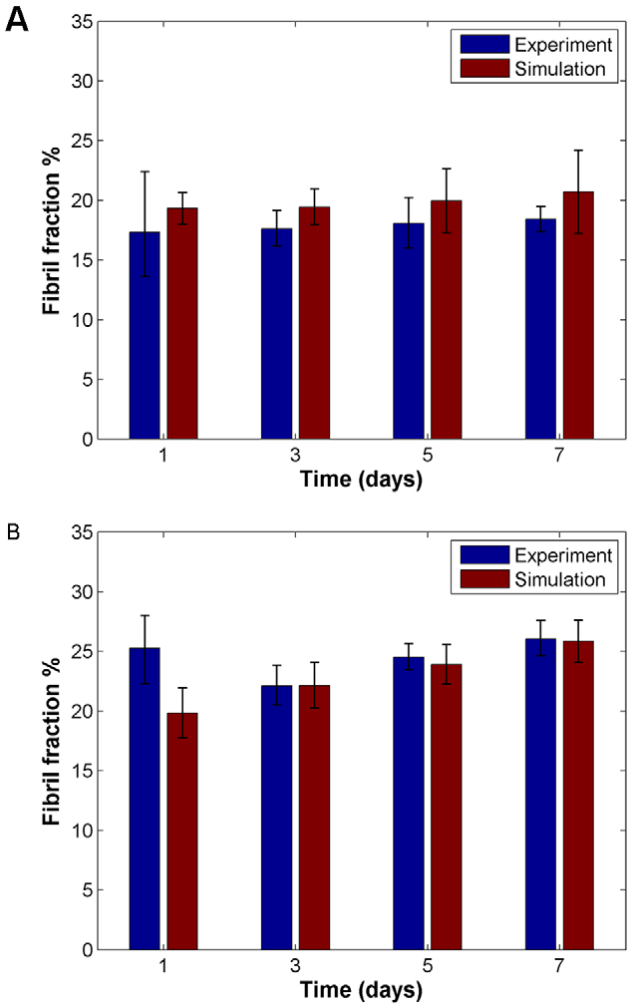
Simulation captures remodeling dynamics. Fibril fractions over time from simulations for (*A*) LNCaP (low invasive) cells and (*B*) DU-145 (high invasive) cells, compared with experimental results, for 3 mg/mL collagen condition. Experimental data was originally reported in [17].

To model the behavior seen in the two prostate cancer cell lines studied in previous studies, we fit model parameters to match our simulated output to experimental data reported in [17]. The parameter values used to fit the remodeling by LNCaP cells and DU-145 cells for the gels of varying collagen content is shown in **Table 1**. The DU-145 cells, the more invasive of the two cell lines [18]–[20], had higher values for parameters that align with increased invasion (P_degMax_ and threshold for local fiber density allowing motility). A direct comparison of the data from the simulated matrices and the experimental data for the two different cell lines in 3 mg/mL collagen over time is shown in **Figure 5** to show how our model's ability to capture the dynamics of remodeling behavior. For both the LNCaP cells (**Figure 5a**) and the DU-145 cells (**Figure 5b**), there is good concordance between the observed and simulated data (p>0.05), with the exception of there being a significant difference between the day 1 DU-145 data (p = 0.02535).

**Table 1:** Model parameter values used for different cell lines.

Parameter	Low invasive	High invasive
Number of cells initially in volume of interest	15	15
P_depMax_	0.8	0.8
P_degMax_	0.1	0.2
P_nothing_	0.9	0.8
Threshold number of fibers allowing deposition	20	20
Threshold number of fibers limiting motility	35	50
Minimum number of cells in volume of interest	14	14

**Figure 6:**
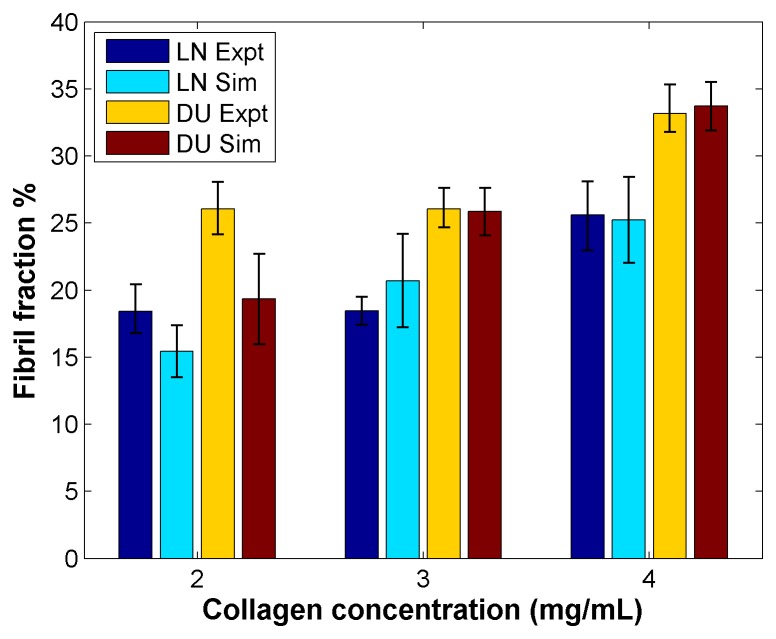
Comparison of results from experiments and simulations across all collagen concentrations. Fibril fractions shown from day 7 for matrices seeded with low invasive and high invasive cells.

Data showing a comparison for the matrices one week after seeding for all conditions is shown in **Figure 6**. We point to **Figure 4** as a baseline to compare how seeding cells in simulated matrices altered the matrix through remodeling in contrast to the “no cell” condition. We were able to successfully replicate the trends of what was seen experimentally using a single set of cellular parameters for each cell phenotype while only altering the number of fibers. There is no significant difference (by a p<0.05 threshold) between simulated and experimental data for a given cell and collagen concentration, with the exception of the DU-145 cells in 2 mg/mL collagen (p = 0.02535). This discrepancy at the lowest collagen concentration may be due to an underestimate of how much collagen deposition may occur since the same cap was applied across all conditions, as only the frequency with which collagen may be deposited, and not the quantity that may be deposited at each time step, was varied with fibril density. Meanwhile, the fibril fractions between the two cell lines differed significantly experimently (p<0.05) for 2 and 3 mg/mL comparisons, and in simulations (p<0.01 for 3 and 4 mg/mL comparisons, p<0.05 for 2 mg/mL comparison). In summary, we note that our model is successful at matching what is seen at various collagen densities.

Our simulations suggest that increased invasiveness results in denser matrices, as was predicted in previous experimental results. Consistent with the experiments we note that in our simulations the more invasive cells deposit more fibers in the low density, or 2 mg/mL, condition (p = 0.01193) while in matrices with higher fiber density, the less invasive cells reduce fibril fraction more dramatically than the high invasive cells (p = 0.009023). These results show that by simply changing two parameters related to invasiveness, we are able to account for what we observed experimentally, corroborating our previously made hypothesis that having increased MMP activity may result in cells residing in a more densely fibrous environment [17].

### Model predictions have been validated by experiments

**Figure 7:**
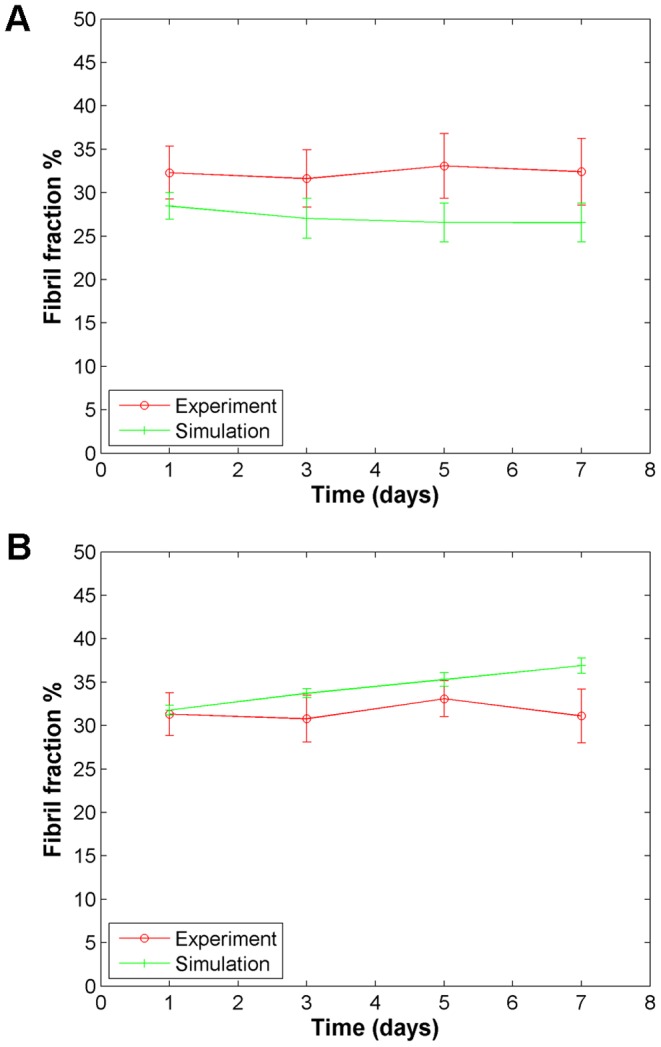
Model validation. Comparison of predicted and observed remodeling behavior for both (*A*) low invasive; and (*B*) high invasive cells, in 4.5 mg/mL collagen over time.

To validate the model, we simulated how the two different cell types, using the cellular parameters obtained before (**Table 1**), modified matrices of higher collagen density (4.5 mg/mL) over one week. We initialized the matrices with 3,500 fibers to match the observed structural properties of 4.5 mg/mL collagen acellular matrices (data not shown). Experiments were then performed, performing CRM at two day intervals over one week to image 4.5 mg/mL collagen matrices seeded with LNCaP cells and DU-145 cells. The resulting experimental and simulation data are compared directly in **Figures 7a and 7b**, and show good agreement, indicating that the model is able to accurately predict the matrix remodeling behavior of these two cell types in 3D collagen environments. It is also important to note that both DU and LN cell lines in high density environments do not migrate much, as shown by other studies [32]; as a result, the overall behavior as seen in Figure 7 is relatively unchanged as a function of time. That is consistent with other studies at higher matrix concentrations [32]. Simulated fibril fractions for the LNCaP-seeded matrices compared to the DU-145-seeded matrices are significantly different for each time point (p<0.01). It is interesting to note that the experimental results for both cell types in this high density collagen converge, suggesting that at higher collagen concentrations, cellular invasiveness becomes a less dominant factor in determining how the matrix is remodeled. The simulation shows different trends over time for the two cell types, with increasing fibril fraction with the less invasive cells and decreasing fibril fraction with the more invasive cells, in contrast to what was seen at the intermediate collagen concentration (**Figure 5**). This indicates that our model does not merely recapitulate the same trends as a function of collagen concentration; rather, that it is sensitive to matrix properties as well.

## Discussion

While matrix remodeling is a fundamentally important physiological process, there have been few comprehensive quantitative experimental studies of the phenomenon under normal and pathological conditions. It is also an area in which there has been relatively little modeling done, with most of the currently existing models focusing on molecular or tissue-level alterations, often in the context of a specific disease. In this paper, we have reported the development and validation of a novel 3D lattice Monte Carlo model of an intermediate scale that explicitly defines both cellular and matrix components, and simulates the effects of collagen fiber density on the remodeling behavior of two cell phenotypes of differing invasiveness.

Using our model, we were able to recreate acellular matrices *in silico* that closely replicated 3D collagen gels, an often-used *in vitro* model system. By varying the number of fibers in our lattice, we were able to qualitatively and quantitatively capture the structure of what is observed when gels are fabricated with different concentrations of collagen. Our model also accounts for both amoeboid and mesenchymal forms of single-cell migration. Cells are allowed to squeeze through extant gaps by realigning fibers to push through in an amoeboid manner (though cell shape is held constantly spherical), and cells are also allowed to migrate in a proteolysis-dependent mesenchymal fashion, degrading a path for movement. Furthermore, we were able to reproduce the results from experiments using gels of varying collagen content seeded with two cancer cell lines of varying invasiveness [17]. We were able to use one set of parameters to define each cell type, consistent with experiments, and observe divergent behavior when the cells were seeded in matrices of different fiber content. The less invasive cell line, which is in part characterized by the secretion of fewer active MMPs [18]–[20], was found in both models and experiments to favor microenvironments with lower fiber density than a comparable cell line that is more invasive. This is somewhat counterintuitive, since one may think that increased MMP activity would result in increased proteolysis and reduced fibril fraction. However, in agreement with our results, the progression of cancer is clinically associated with both increased MMPs [34] and the stiffening of tissues locally [35], [36]. Increased collagen content correlates with increased elastic modulus [17], [32], [37], and our data indicate that indeed, the more invasive cell line resides in a higher density microenvironment. In breast cancer, for example, regions around tumors are associated with higher collagen density [38], and consequently we are able to diagnose the disease through palpation. This model provides some mechanistic insight into factors that contribute to cancer progression, and may have clinical applications, as one can test how perturbing the different cellular and matrix parameters may affect matrix remodeling behavior as a means of identifying potential therapeutic targets.

Our results suggest that highly invasive cells are more amenable to higher density matrices since they are better equipped to navigate through such environments. More invasive cell lines may indeed benefit from more fibrous environments since collagen can aid in invasion and proliferation [39]. Indeed, it has been recently shown that in glioblastoma multiforme, a highly metastatic type of brain tumor, cancer cells deposit fibrillar collagen, which is not typically found in brain ECM, to facilitate invasion [40]. In contrast, less invasive cells may need to exert more energy in selectively degrading or pulling on fibers using their contractile machinery to create larger pores and do not appreciate a net benefit from such dense microenvironments. Since both cell lines studied reduced the fibril fractions for the highest density collagen condition tested, it appears that even highly invasive cell lines have an upper limit on how dense an environment they are able to grow and thrive in.

It should be noted that while this model is able to capture various experimental observations, it does have a number of limitations. This model was designed to match an *in vitro* experiment, so the structural parameters for the simulated matrices have been tuned accordingly. Elastography studies on clinical samples indicate that normal prostate tissue has an elastic modulus on the order of 50 kPa while cancer prostate tissues are about twice as stiff [36]. In contrast, the gels prepared for this study ranged from about 250 to 550 Pa, depending on collagen concentration [17], suggesting that they are much less dense than clinical samples since collagen is the main tensile component of ECM. An investigation of collagen content from prostate biopsy samples showed that normal and cancerous prostate tissue is about 20–30% collagen by weight, with the highest collagen content seen in samples from more advanced tumors [41]. However, it is difficult to correlate these weight percentages to collagen concentrations used in culture experiments since *in vivo*, the tissue would have been hydrated, just as the 3D collagen gels we use in experiments are. Observations that cells can align collagen fibers on a large scale [38], [42], [43] also cannot be captured by the model given its stochastic nature. If parallel organization of fibers were favored, directed cell migration along the axis of fiber orientation would likely be preferred due to reduced steric hindrance along that path. Matrix remodeling therefore may be focused in that area, rather than randomly distributed throughout the whole lattice. Currently, the model does not account for any other ECM components except for collagen, which is adequate for capturing the experimental system used in various experimental studies but may not be sufficient for modeling tissues and more complex environments. Cells in the model also maintain a spherical geometry when *in vitro* and *in vivo*, cells adopt a more spindle-like morphology. Furthermore, the simulation, as it currently stands, does not account for the proliferation or apoptosis of cells, though these may alter the overall remodeling dynamics. As a purely Monte Carlo model, the simulation ignores the potentially significant effect of growth factors and the contribution of other autocrine and paracrine factors [44]. Finally, many assumptions were made on the probability distributions and parameter values (as discussed in the modeling section) since as of yet, there is no way to obtain concrete numbers for a number of parameters from experiments. However, with this report, we have demonstrated that by fitting the model to experimental data, we are able to obtain parameter values that are reasonable given what we know about the cell lines used in the original study. Using these same cellular parameters, we were also able to accurately predict the remodeling behavior of these cells in environments of varying collagen content. To predict the behavior of other cell lines, it would be important to know their relative invasiveness and proteolytic activity. In principle, reasonable values based on what we have presented here could be determined by comparing the relative motility (e.g. by looking at cell speeds) and proteolytic activity (e.g. by zymography) of different cells lines compared to that of the LNCaP cells and DU-145 cells used here. However, to validate the parameter value selection, fitting to experimental results would be necessary.

Our model is able to capture both qualitative and quantitative features of experimental results. The power of this model lies in its ability to explicitly define properties for both the matrix and cells, which is important since the interplay between the two drives the remodeling behavior observed [17], [44]. The model allows for the modulation of cell size, density, motility, and propensity for proteolysis, as well as matrix fiber density and orientation with minimal effort, making it an attractive platform to study a variety of different biological contexts. This systems-level type approach to understanding matrix remodeling is in contrast to other models, such as [45] which focuses narrowly on the effects of MT1-MMP turnover on ECM degradation.For one, our model can be used to predict how seeded cells may modify biomaterial constructs in tissue engineering applications. Alternatively, the model may be used to simulate the effect of co-culture, which is of interest when attempting to studying systems with hetereogeneous cell populations as in cancer or complex tissues, by seeding the lattice with two or more distinct cell populations defined by different parameters. The model can also be used to investigate different disease states that involve ECM remodeling, such as atherosclerotic plaques [46], by allowing for the description of a complex 3D matrix structure seeded with diverse cell populations. It should be noted, however, that 3D systems are not always physiologically relevant and selecting appropriate matrix and cellular parameters would be critical in obtaining results with any predictive power at all. Overall, this work may provide significant, clinically-relevant insights into the many different processes in which matrix remodeling plays a critical role.

## Methods

We used a lattice Monte Carlo approach to study ECM remodeling in 3D. Our focus was to develop a novel model and create a computational platform that would enable us to directly compare our results to experiments. In order to do so, we simulate cells on a volume of interest that is defined by a 50×50×50 3D lattice with 4.5 µm spacing between nodes, and the ECM fibers on a larger lattice (75×75×75), centered on the cellular lattice. We simulated the cells on the smaller lattice in order to eliminate edge effects.These dimensions are selected to match the dimensions of xy-slices taken with CRM experimentally [17]. The cell density selected of 14 to 15 cells per lattice corresponds to a seeding density of about 400,000 cells/mL, which was reasonable given that the initial seeding density of experimental gels was 200,000 cells/mL and that proliferation was not allowed in the model. The timestep for the simulation is 30 minutes, a compromise between the dynamics of cell motility (cell speeds are typically reported in µm/hr) and collagen remodeling (pulling of individual fibers can occur in minutes [47]). This is also consistent with previous studies on cell migration [48]. To initialize the matrix, *x* cells, represented by spheres of radius 9 µm (consistent with previous Monte Carlo models of LNCaP cells [49]), are first randomly positioned on the lattice, maintaining volume exclusion between cells. The lattice is then filled with *y* collagen fibers, represented by line segments. To position the fibers, one end of each segment is first randomly positioned on the lattice. The second end of each fiber then is defined via spherical coordinates, with randomized orientation, selecting elevation and azimuth angles such that 0≤θ≤π and 0≤φ<2π respectively, and length constrained to a distribution of fiber lengths observed experimentally [17]. In our previous experiments, changing collagen concentration only significantly altered the number of fibers in the gels, with the distributions of fiber diameters and lengths remaining fairly consistent [17], and thus the distributions were not changed for different collagen concentrations. Volume exclusion between the fibers and cells is strictly enforced, but fibers are allowed to intersect and overlap to more closely replicate the complicated networked ECM structure seen *in vitro* [12], [13], [17] and *in vivo* [50]. As the simulation evolves in time, the cells and fibers are not constrained to the lattice nodes and are free to move about in the volume.

At each time step, the model iterates through each cell. As described earlier, each cell is allowed to move with some probability P_move_ that is a function of the number of fibers within a specified radius of each cell. Literature suggests that it is unclear just how far cells can sense in the matrix, though some work has indicated that strains can be detected on length scales on the order of nanometers [51]. We chose a radius of 3 lattice units, or 50% more than the cell radius, as a reasonable distance over which a cell may probe the local microenvironment over a time step by extending out protrusions. Recent reports on collagen remodeling during angiogenesis showed fibril organization occurring dynamically tens of microns ahead of sprouting endothelial cells [52], indicating that cells are capable of directing matrix remodeling from distances on the order of cell lengths away. When the number of close fibers is above some threshold, movement is not allowed to simulate the effects of steric hindrance. The likelihood of movement is implemented with a Gaussian curve to model the balance between traction and adhesion forces observed experimentally [30]–[32]. A cell moves to a position in a random direction up to 2 lattice units, or 9 µm away, corresponding to a maximum cell speed of 18 µm/h, which is consistent with experiments of cell migration in 3D [37], [53]. Since it is unlikely for a cell to randomly find a space in the fibrous network large enough to accommodate it, migration in our simulation is accompanied by matrix remodeling by means of matrix deformation and proteolysis, as has been extensively reported experimentally [1], [54]. In our model, fibers that intersect with a cell's new position can be dealt with in one of two ways: the fiber may be degraded, which for simplicity consists of erasure from the system, or the fiber may be pulled by the cell. Fibers are more likely to be pulled on than degraded, a bias that was implemented due to the experimental observation of wide-scale contraction of collagen gels [55]. If the number of fibers that intersects with a cell's new position is above some threshold, that new position is determined to be excessively sterically hindered and another position is randomly selected up to 2 units away from the cell's previous position and retested. Volume exclusion between other cells is also enforced.

The proximity of each fiber to each cell is tested as well. When it is within a specified radius of a cell, a fiber is identified as close and eligible for remodeling by the cell. Cells that move in a given timestep however are not allowed to modify any more fibers. The number of fibers neighboring each cell, whether it has moved or not, is updated at each timestep to calculate the probabilities for different remodeling behavior for the next timestep. The probability P_degrade_ is used to determine if a close fiber will be degraded by a cell that has not moved. This probability is a logistic function of closeF, the number of fibers neighboring a given cell in the previous timestep, multiplied by a maximum likelihood of degradation, P_degMax_, such that:
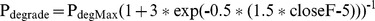
This logistic function was selected such that there would be a high probability of fiber degradation in the presence of a high local fiber density and a low probability of fiber degradation in the presence of a very low fiber density. There is also a probability that nothing will happen to each close fiber, P_nothing_, and a probability of fiber realignment, P_align_ = 1−P_nothing_−P_degrade_. When a fiber is realigned, the end farther from the cell is moved, changing the orientation (with a specified maximum degree of orientation change to preclude unrealistically extreme changes) while maintaining the fiber's length. Moving the distal end replicates how a cell can pull on a fiber using its actomyosin machinery as observed in other studies [47]. With fiber alignment, it is possible that the fiber's new position will intersect with a cell. To address this problem and maintain volume exclusion between cells and fibers, the alignment algorithm selects a new orientation for the fiber of interest randomly from a region with the most possible allowable new, non-cell-interfering positions for the fiber. Once a fiber has been degraded or realigned, it cannot be modified by another cell in a given timestep. If the number of fibers close to a cell falls below a certain threshold, there is a probability P_deposit_ of new fiber deposition, again a logistic function dependent on local fiber density multiplied by a maximum likelihood of deposition, P_depMax_, similar to the expression for P_degrade_. New fibers are placed close to the source cell, and are again of random orientation and length. Each cell can only deposit one fiber at each timestep. At the end of each timestep, the fibers marked for degradation are erased and new fibers from all the cells are added to the system.

To offset the effect of migration of cells outside of the 50×50×50 lattice over time, cells are randomly added to the edges of the volume of interest when the number of cells within the lattice falls below some minimum number to maintain a near constant cell density. Fibers are actually simulated over a larger lattice to avoid cells migrating preferentially towards the lattice edges where there are fewer fibers. Only cells (and therefore their neighboring fibers) within the 50×50×50 region of interest are tracked and evolving with time. This model therefore simulates what is occurring in a subsection of a larger matrix structure, such as the collagen gels studied in our previous experimental paper [17].

To quantify how much matrix remodeling is occurring, the model outputs the fibril fraction of the 3D matrix at specified time intervals. To obtain fibril fraction, the same image processing procedure used in our experimental paper is performed on the simulated dataset [17]. The image processing is performed using ImageJ (NIH, Bethesda, MD), MIJ (EPFL, Lausanne, Switzerland), and Matlab (The MathWorks, Natick, MA). Briefly, an xy-slice of the 3D lattice is taken and binarized in ImageJ, leaving the fibers as black pixels. The fraction of the area occupied by fibers is measured (“fibril fraction”) and taken as a metric of the fiber density in the matrix as it evolves in time, similar to what other groups had done [33].

The simulation was implemented in Matlab (The MathWorks, Natick, MA). The model was run for 336 time steps for one week of biological time, to replicate the experiments. Different levels of seeded cell invasiveness were modeled by using different sets of maximum probabilities, probability distributions, and thresholds. General model parameters are enumerated in **Table 2**. The values were selected based on experimental literature, though in many cases, the data for the thresholds, probability distributions, and maximum probability values specific to our model were unavailable. In those cases, we selected values that were reasonable given our current knowledge and observations from the extensive CRM data sets we have acquired over time (data not shown). In the context of our generalized model, it is useful to think of the cells modeled not as specific cell lines, but as lowly invasive and highly invasive model cell lines. Specific parameters used to model the different cell lines, obtained by fitting values to produce results that matched experimental data, are listed in **Table 1**. This fitting was performed by systematically testing a range of values for each parameter and selecting the values that yielded the best match of simulation outputs to the experimental results taken directly from [17]. For each set of parameters, the simulation is run at least 5 times. Error bars in graphs indicate 95% confidence intervals. The structural data from CRM experiments were not normally distributed so confidence intervals were calculated using the bias-corrected, accelerated bootstrapping algorithm [17]. The Mann-Whitney test was performed to calculate p-values for comparisons using R. The model will be available for collaboration with the broader scientific community in an open source manner.

**Table 2:** Model parameter values and references that guided their selection.

Size of cellular lattice	50×50×50	
Size of ECM lattice	75×75×75	
Space between lattice nodes	4.5 µm	
Length of time step	30 minutes	[32], [47]
Cell radius	9 µm	[49]
Radius around cell defined as “close”	13.5 µm	[52]
Maximum cell speed	18 µm/hr	[37], [53]
Range of fiber lengths	4.5–45 µm	[17]
2 mg/mL collagen	1200 fibers in ECM lattice	[13], [17]
3 mg/mL collagen	2000 fibers in ECM lattice	[13], [17]
4 mg/mL collagen	3000 fibers in ECM lattice	[13], [17]

For validation of the model, experiments were performed on LNCaP- or DU-145-seeded 4.5 mg/mL collagen matrices, as described in our previous work [17]. The concentration of 4.5 mg/mL was selected as a different collagen concentration that was obtainable with our system, as concentrations less than 2 mg/mL yielded excessively fragile matrices, while the density of the stock collagen solution precluded the use of concentrations higher than 4.5 mg/mL. The remodeling data for 2, 3, and 4 mg/mL collagen that is reported here was taken directly from this previous paper [17]. Briefly, the two cell lines (both from ATCC, Manassas, VA) were cultured using standard cell culture techniques. Cells were suspended in 3D type I collagen (BD Biosciences, San Jose, CA) at a final density of 200,000 cells/mL in a solution consisting of an equal volume of collagen and neutralizing solution (100 mM HEPES buffer (Fisher Scientific, Pittsburgh, PA) in 2× phosphate buffered saline (PBS), pH 7.3), as previously reported [56]. Gels were prepared in triplicate.

CRM was performed using a scanning confocal microscope (Olympus FV1000) with a 60×1.2 N.A. water immersion lens, exciting the sample with a low intensity 488 nm laser and collecting signal between 485–495 nm. Three 30 µm stacks with 0.5 µm-thick slices were obtained from randomly selected, cell-containing regions in the gel 1, 3, 5, and 7 days after plating. Image processing of the CRM datasets was performed as described for the simulated matrices.
